# A dynamic Boolean network reveals that the BMI1 and MALAT1 axis is associated with drug resistance by limiting miR-145-5p in non-small cell lung cancer

**DOI:** 10.1016/j.ncrna.2023.10.008

**Published:** 2023-10-19

**Authors:** Shantanu Gupta, Daner A. Silveira, Gabriel P.S. Piedade, Miguel P. Ostrowski, José Carlos M. Mombach, Ronaldo F. Hashimoto

**Affiliations:** a Instituto de Matemática e Estatística, Departamento de Ciência da Computação, Universidade de São Paulo, Rua do Matão 1010, 05508-090, São Paulo, SP, Brazil; b Children's Cancer Institute, Porto Alegre, Rio Grande do Sul, Brazil; c Departamento de Física, Universidade Federal de Santa Maria, Santa Maria, 97105-900, RS, Brazil

**Keywords:** Drug resistance, NSCLC, MALAT1, miR-145, Positive feedback loops, BMI1, Apoptosis

## Abstract

Patients with non-small cell lung cancer (NSCLC) are often treated with chemotherapy. Poor clinical response and the onset of chemoresistance limit the anti-tumor benefits of drugs such as cisplatin. According to recent research, metastasis-associated lung adenocarcinoma transcript 1 (MALAT1) is a long non-coding RNA related to cisplatin resistance in NSCLC. Furthermore, MALAT1 targets microRNA-145-5p (miR-145), which activates Krüppel-like factor 4 (KLF4) in associated cell lines. B lymphoma Mo-MLV insertion region 1 homolog (BMI1), on the other hand, inhibits miR-145 expression, which stimulates Specificity protein 1 (Sp1) to trigger the epithelial–mesenchymal transition (EMT) process in pemetrexed-resistant NSCLC cells. The interplay between these molecules in drug resistance is still unclear. Therefore, we propose a dynamic Boolean network that can encapsulate the complexity of these drug-resistant molecules. Using published clinical data for gain or loss-of-function perturbations, our network demonstrates reasonable agreement with experimental observations. We identify four new positive circuits: miR-145/Sp1/MALAT1, BMI1/miR-145/Myc, KLF4/p53/miR-145, and miR-145/Wip1/p38MAPK/p53. Notably, miR-145 emerges as a central player in these regulatory circuits, underscoring its pivotal role in NSCLC drug resistance. Our circuit perturbation analysis further emphasizes the critical involvement of these new circuits in drug resistance for NSCLC. In conclusion, targeting MALAT1 and BMI1 holds promise for overcoming drug resistance, while activating miR-145 represents a potential strategy to significantly reduce drug resistance in NSCLC.

## Highlights

• A Dynamic Boolean model uncovers the role of miR-145 in drug resistance in NSCLC, targeting MALAT1 and BMI1.
• Novel positive circuits involving miR-145-5p identified in drug resistance regulation.
• miR-145's intricate interplay with Sp1, Myc, KLF4, and Wip1 contributes to drug resistance in NSCLC.
• Perturbation analysis identifies potential therapeutic strategies to overcome drug resistance in NSCLC.
• miR-145 emerges as a key player in NSCLC drug resistance modulation.

## 1 Introduction

Current studies suggest that non-coding RNAs (ncRNAs) play a significant role in the modulation of drug resistance by downstream DNA damage pathways. In this setting, Cui et al. [1] revealed that MALAT1 overexpression directly targets miR-145 and controls KLF4 expression, which is implicated in drug resistance in NSCLC cells. More evidence comes from an investigation by Chang et colleagues [2], who showed that when BMI1 is overexpressed, miR-145 is downregulated, which boosts Sp1 expression and triggers EMT in pemetrexed-resistant NSCLC cells [2]. Interestingly, the expression of MALAT1 is induced by Sp1 in NSCLC [3]. On the other hand, additional NSCLC research showed that overexpression of miR-145 might block cell cycle machinery at the G1/S checkpoint by inhibiting Myc [4] and Bcl2 [5], which in turn prevented NSCLC from proliferating by inducing cell cycle arrest and apoptosis [4,5]. LncRNAs are ncRNAs with a length of over 200 nucleotides. LncRNAs have a variety of roles in biology, including cell-cycle progression, apoptosis, and genomic stability [6]. Similarly, miRNAs, which typically range from 19 to 25 nucleotides in size, play various roles in biological systems, including their involvement in tumor formation [7].

The objective of modeling a complex system, such as the MALAT1/miR-145 axis in drug resistance, is to produce a model that can predict the outcome of each component quantitatively. Therefore, Boolean network modeling (BNM) is the most effective way for integrating available knowledge into a logical framework that is compatible with experimental data [[8], [9], [10], [11]]. Signaling molecules (signaling proteins and noncoding RNAs like lncRNAs and miRNAs) are described as nodes, and the interconnections between them are called edges [[12], [13], [14], [15], [16], [17], [18]]. Cell outcomes are coupled to model attractors (endpoints or cyclic attractors), whose characterization and reachability features are well suited to this approach [19]. Additionally, analyzing closed pathways (parallel to feedback loops in the continuous model) linking the two or more nodes in a network may also function as regulatory circuits influencing the dynamics of the network, which is another characteristic of BNM [[20], [21], [22]]. For more details about BNM, refer to the Methods section.

**Fig. 1:**
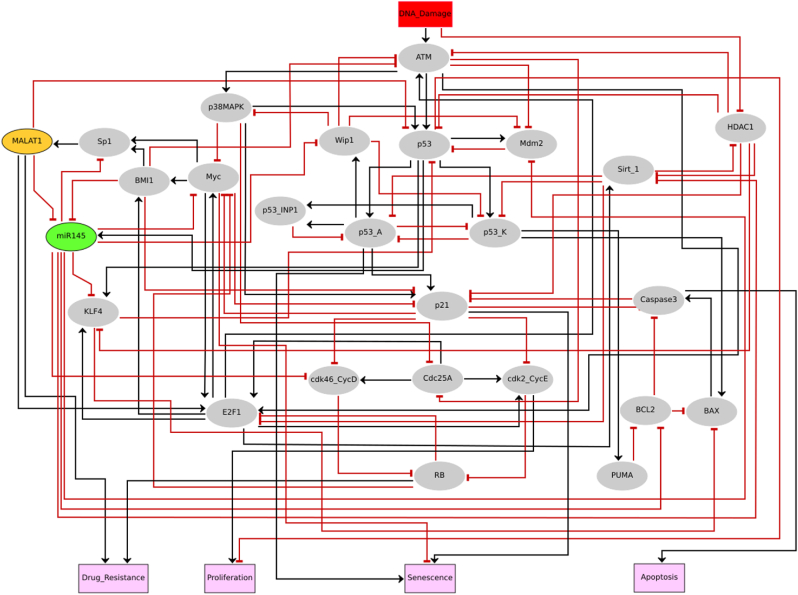
**The network of drug resistance involving MALAT1/miR-145 axis in NSCLC**. Direct black edges that end in an arrowhead indicate positive interactions or regulatory relationships. Direct red edges that end in a hammerhead indicate negative interactions or regulatory relationships. The color of the nodes represents their function, as follows: signaling proteins are elliptic (gray nodes), lncRNA MALAT1 in an elliptic node (orange), and miR-145 in an elliptic node (green). The input rectangle node in red represents DNA damage. The outputs of the model, shown in pink at the rectangular nodes, include drug resistance, proliferation, senescence, and apoptosis. The full names of network components corresponding to each node and biological justification for the edges with their regulators are provided in Supplementary Table S1.

Inspired by the above experimental observations; we propose a dynamic Boolean network of the MALAT1/miR-145 axis associated with drug resistance in NSCLC (see Fig. 1).

## 2 Methods

### 2.1 The topology of the gene regulation network in NSCLC cells and the combination of public databases/tools

Only PubMed articles and databases like BIOGRID 3.5 (https://thebiogrid.org) [23] were used to construct a gene regulatory network for ncRNAs (MALAT1 and miR-145). The objective was to identify genes or proteins that were targeted by these ncRNAs (Fig. 1). For example, the targets of miR-145 such as Cyclin-dependent kinases 4 and 6 complexes/CyclinD1 (CDK4/6-Cyclin D) [4], Mdm2 [24], Sirt1 [25], Myc [4], Sp1 [2], KLF4 [1], and BCL2 [5], have been studied extensively. Whereas, MALAT1 targets miR-145 [2] and p53 [26]. We employed public datasets for this, such as TARGET SCAN HUMAN 7.2 (http://www.targetscan.org/vert_72/) [27].

The Boolean model was built, simulated, and the results were shown by GINsim 3.0.0b (http://www.ginsim.org/downloads) [28]. GINsim algorithms detect all attractors in both the wild-type systems (unperturbed Boolean model) and mutant instances. The model file may be accessed from the "Code Availability" section.

### 2.2 Dynamic Boolean network model, rules, and simulations based on PubMed literature

The Boolean technique is grounded in the examination of a regulatory graph, whereby every node represents a signaling component and each straight edge (or arc) represents an activation or inhibition between two nodes. Nodes are Boolean variables that only allow "0" and "1" values, corresponding to "active" and "inactive" states. Each node in the network is given a logical rule based on the interpretation of the biochemical information, which governs its activation level in relation to the location of its regulators [29].

The biological interconnections stated in the gene regulatory network (Fig. 1) were encoded into Boolean rules to establish a Boolean network of ncRNAs (MALAT1 and miR-145). These Boolean rules for governing nodes are based on PubMed biological literature and may be found in Supplementary Table S1. The classic Boolean operators "AND," "OR," and "NOT" were employed to build these rules. The key outcome of simulations utilizing a Boolean network is attractors. A state transition graph (STG) allows us to know the dynamical functioning of a Boolean model. Every node in this graph reflects the present state of the network variables, and the arcs describe conversions between these states. The STG accommodates all potential trajectories from such an initial state to a final state. Stable states (or fixed points) are terminal nodes with no outgoing edges, whereas a cyclic state is considered a series of transitions locked within a fixed group of states in the STG. Asynchronous updates were considered to account for state updates, which may reflect the non-deterministic behavior exhibited in molecular networks [20]. Additionally, negative and positive circuits (also known as feedback loops) govern the dynamics of a gene regulatory network. Negative circuits can stimulate oscillations, while positive circuits are in charge of multi-stable dynamics. Furthermore, this method allows for in silico gain-of-function (GoF) or loss-of-function (LoF) perturbations, in which we constrain node values to be "active" or "inactive", respectively. This method makes it easier to investigate the influence of individual nodes on network dynamics and the resulting phenotype [28,29].

### 2.3 Model mechanism for drug resistance in NSCLC

In our previous study on non-small cell lung cancer (NSCLC) [21], we provided insights into the role of miR-34a in the modulation of p21-dependent senescence and apoptosis. We demonstrated that ATM activation of miR-34a contributes to the regulation of these cellular processes in NSCLC. Specifically, p21, an inhibitor of apoptosis through its inhibitory effect on caspase 3, is influenced by various factors in NSCLC. Myc and HDAC1 inhibit p21, while miR-34a directly suppresses Myc and HDAC1, leading to p21 activation and subsequent induction of senescence. The activation of p21 serves as a crucial switch between senescence and apoptosis in NSCLC. For a more detailed understanding, we refer readers to our previously published paper [21]. We employed a previously published model in our investigation to uncover the role of these ncRNAs in drug resistance mechanisms in NSCLC and extended it through the miR-145 and MALAT1 axis. Therefore, we define the interactions which are associated with the miR-145/MALAT1 axis.

It is well known that p53 promotes miR-145 expression [30]. When miR-145 is activated, it targets Myc [4], Cyclin D [4], Bcl2 [5], Sirt1 [25], Sp1 [2], KLF4 [1], and MDM2 [24]. Myc directly activates BMI1 expression [31] and upregulated BMI1 directly inhibits miR-145 [2] and forms a positive circuit between miR-145 (miR-145/Myc/BMI1), additionally, it triggers Sp1 [2] and KLF4 expression [1]. In turn, upregulated Sp1 activates MALAT1 [3], upregulated MALAT1 directly inhibits miR-145 [1], whereas upregulated KLF4 inhibits p53 [32], form two positive circuits between them (miR-145/Sp1/MALAT1 and miR-145/KLF4/p53). Moreover, BMI1 directly inhibits ATM [33] and p21 [34]. Furthermore, upregulated MALAT1 directly target miR-145 [1] and p53 [26]. Additionally, p21 and Caspase 3 are interlinked through a positive circuit, reinforcing their connection [35]. Similarly, Sirt1 and HDAC1 are tethered by a positive circuit, highlighting their interdependence [36]. At the G1/S checkpoint, the inhibition of cyclin D by CDK4/6 and of cyclin E by CDK2 promotes RB1 expression, a critical step in blocking the G1/S transition [37]. E2F1 exhibits interactions with both Myc [38] and ATM [39], signifying its versatile role in cellular processes. Myc also engages with p21 [40], further underscoring its involvement in the G1/S checkpoint. Notably, p53 plays a pivotal role in G1/S checkpoint activation [41]. Myc and E2F1 participate in multiple positive circuits involving p21, RB, and cyclins [[42], [43], [44]]. Conversely, p53 is implicated in various negative circuits, which include interactions with KLF4 [45], Mdm2 [46], Wip1 [47,48]. Additionally, E2F1 and Sirt1 are linked through a negative circuit [49], adding to the intricate regulatory landscape [49].

By employing the biochemical data provided above, we propose a BNM for drug resistance in NSCLC cells by controlling the function of miR-145 into consideration.

## 3 **Results**

### 3.1 Development of a BNM and its endpoints

The network contains 27 signaling components, including one lncRNA (MALAT1) and one miRNA (miR-145). A single input called DNA damage is also included and has two possible states: "ON" and "OFF." Moreover, there are 85 direct interconnections between these signaling components. Our Boolean model is based on published data for an NSCLC cell line, primarily derived from four experimental investigations [1,2,4,5]. Additional experimental studies can be found in Supplementary Table S1.

**Fig. 2:**
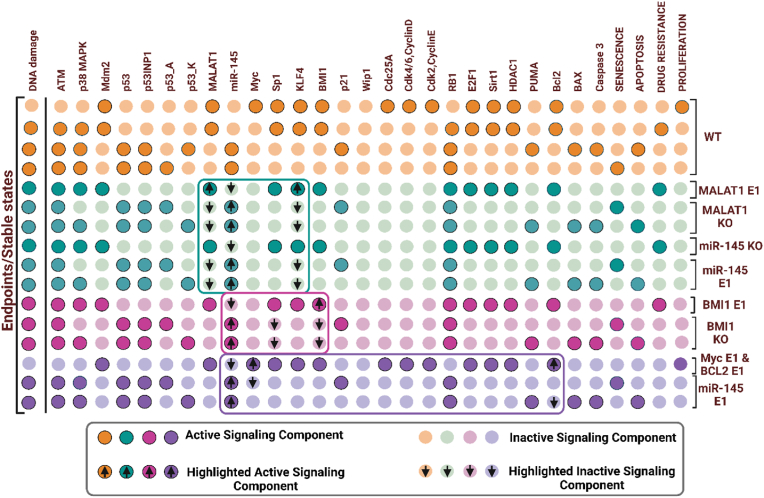
**Wild-type system of the network and experiments support to gain or loss-of-function perturbations of the model.** Each line represents an endpoint (also known as a stable state). Orange circles reflect the network's wild-type system. Green, magenta, purple, and red circles, on the other hand, appropriate balance of skills in experimental observations. In more detail, light color circles of orange, green, magenta, purple, and red represent inactivation. Dark color circles of orange, green, magenta, and purple, on the other hand, signify activation. Green circles represent MALAT1/miR-145/KLF4 axis by Cui et al. [1]. While magenta circles represent BMI1/miR-145/Sp1 axis by Chang et al. [2]. Purple circles are shown from the studies of Chen et al. [4] and Pan et al. [5], respectively.

The wild-type system of the model has four endpoints (see Fig. 2 in orange circles), which are as follows: First, in the absence of DNA damage (input), the endpoint drives proliferation through the activation of cell cycle promoters, including Myc, Sp1, Cdk, and cyclins. Second, in the presence of DNA damage, the drug resistance endpoint is triggered, due to the activation of MALAT1, SP1, and KLF4, but p53 and miR-145 are not activated, allowing cells to develop resistance to treatment. Third, p53-A/p21 promotes senescence, a state of growth arrest to prevent damaged cells from dividing further. Finally, fourth, p53-K/BAX induces apoptotic cell death, thereby eliminating defective cells. For more details, see Fig. 2 in orange circles.

### 3.2 BNM validation through the experimental observations from NSCLC

To determine if the MALAT1 and miR-145 may influence cell proliferation as reported by the findings summarized in Table 1. We chose to execute Gain or Loss-of function perturbations of the corresponding component. Initiated with the investigation by Cui et al. [1]. Cui and colleagues found that by activating KLF4, MALAT1 overexpression (E1) downregulates miR-145 expression and accelerates drug resistance, as seen in Fig. 2 (highlighted in the green circles). In contrast, MALAT1 knockdown (KO) triggers the overexpression of miR-145, which targets KLF4, leading to improved drug sensitivity [1]. Similarly, silencing miR-145 improves drug resistance. While overexpression (E1) of miR-145 prevents drug resistance. Likewise, Chang and colleagues [2] uncovered overexpression of BMI1 (E1) reduces miR-145 expression, which activates Sp1 and promotes drug resistance (Fig. 2), highlighted in the magenta circles). However, knocking it out increases the expression of miR-145, which inhibits Sp1 and so boosts drug sensitivity (see Fig. 2, emphasized in the magenta box). After that, Chen et al. [4] showed that Myc is upregulated (E1) in NSCLC cells and that miR-145 directly targets Myc and Cdk4,6/cyclin D in response to DNA damage, inhibits tumor growth, and induces senescence (see Fig. 2, underlined in the purple circles).

**Table 1:** Validation of model using experimental investigations.

Experimental observation	Experimental technique	Cell lines	References
MALAT1 suppresses miR-145 and promotes the activation of KLF4 and induces Cisplatin resistance.	*In-Vitro*	A549, H1299	[1]
BMI1 suppresses miR-145 and stimulates Sp1 expression and induces Pemetrexed resistance.	*In-Vitro*	A549, A400	[2]
Overexpression of miR-145 inhibits BCL2-induced apoptosis.	*In-Vitro*	A549	[5]
Overexpression of miR-145 inhibits Myc induced cell cycle arrest.	*In-Vitro*	A549	[4]

Similarly, Bcl2 was found to be increased in NSCLC cell lines by Pan et al. [5]. In response to DNA damage, miR-145 directly targets Bcl2, which activates BAX/Caspase-3 and induces apoptosis. As seen in Fig. 2, our model agrees well with the experimental observations [1,2,4,5].

### 3.3 Feedback loops and analysis

Gene regulatory networks (GRNs) are constituted from both positive and negative circuits. These circuits are capable of capturing and analyzing the dynamics of biological systems. To determine whether our network is capable of forming some circuits, we choose to inquire about them.

GINsim only uncovered 30 genuine biological circuits that actively alter network dynamics. Nevertheless, we only choose 21 of them, from a total of four components, as several of them had previously experienced experimental confirmation (see Table 2).

**Table 2:**
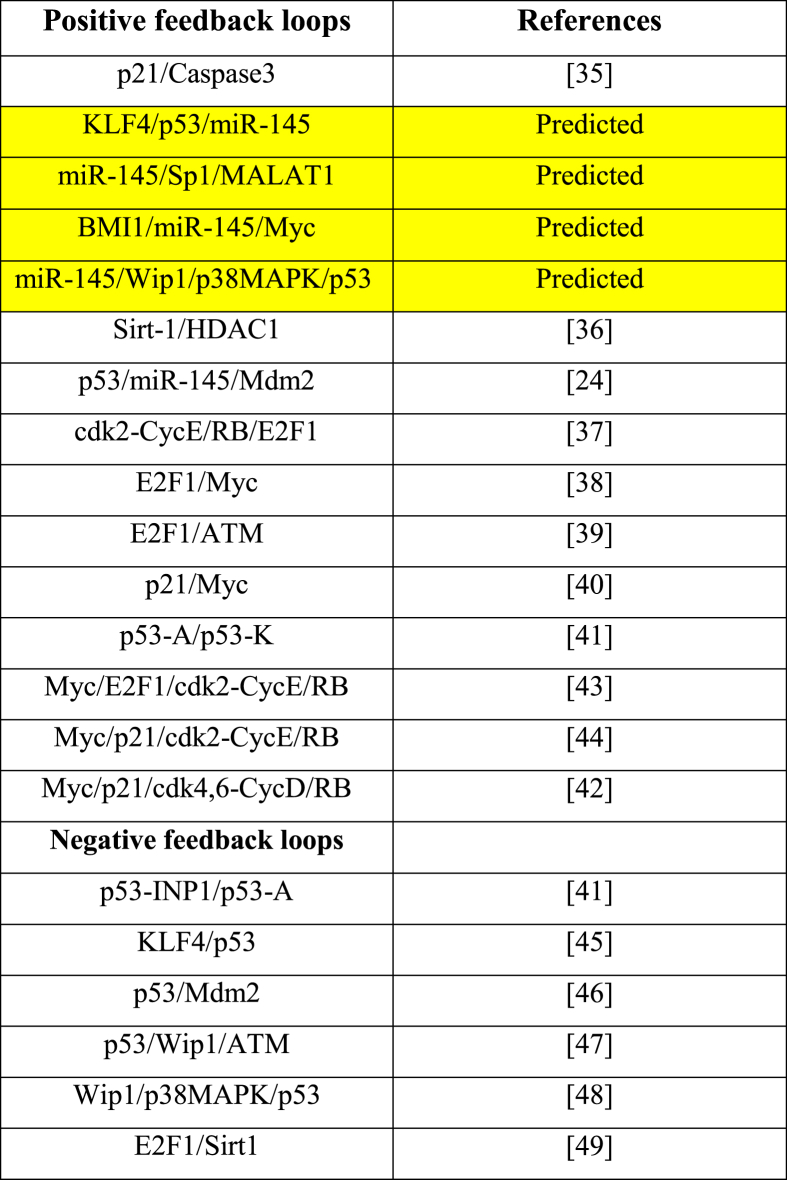
**Boolean network functional circuits and experimental obedience.** The network's predicted positive circuits are highlighted in yellow.

Four of these 21 circuits are unique (see Table 2, emphasized in yellow), including miR-145/Sp1/MALAT1, BMI1/miR-145/Myc, KLF4/p53/miR-145 and miR-145/Wip1/p38MAPK/p53. We opted to investigate these four novel circuits exclusively because we wanted to verify the effectiveness of the MALAT1/miR-145 axis in drug resistance in NSCLC. Interestingly, the molecular interactions that distinguish this circuitry for NSCLC cells are known in the literature (Table 3); nevertheless, their functional implications in drug resistance and cellular responses to chemotherapy in NSCLC may still be unknown or poorly understood. By focusing on these circuits in the context of NSCLC drug resistance, we intend to uncover novel insights and potential therapeutic targets that may have been ignored in prior investigations. In this way, analyzing these circuits in the context of NSCLC drug resistance may lead to novel insights, potentially uncovering regulatory mechanisms or targets that have not been previously considered.

**Table 3:** The literature is replete with evidence of biochemical interactions that characterize a biological positive circuit.

Positive Circuit	Circuit Elements	Target	Direct Interaction	References
miR145/Sp1/MALAT1	miR-145	Sp1	Direct inhibition	[2]
	Sp1	MALAT1	Direct activation	[3]
	MALAT1	miR-145	Direct inhibition	[1]
BMI1/miR-145/Myc	BMI1	miR-145	Direct inhibition	[2]
	miR-145	Myc	Direct inhibition	[4]
	Myc	BMI1	Direct activation	[31]
KLF4/p53/miR-145	KLF4	p53	Direct inhibition	[32]
	p53	miR-145	Direct activation	[30]
	miR-145	KLF4	Direct inhibition	[1]
miR145/Wip1/p38MAPK/p53	miR-145	Wip1	Direct inhibition	[50]
	Wip1	p38 MAPK	Direct inhibition	[48]
	p38 MAPK	p53	Direct activation	[48]
	p53	miR-145	Direct activation	[48]

Therefore, we attempted to explore whether these four circuits were effective for drug sensitivity in NSCLC. We employed circuit component perturbation to establish which circuits are responsible for drug resistance according to phenotype occurrence. Thus, we construct Table 4, which summarizes the findings. As we can see in Table 4, when we obtained a specifically apoptotic phenotype, we considered it the most convincing argument to reduce drug resistance, which we highlighted in green cells. Still, enhanced drug resistance is associated with senescence and drug resistance phenotypes, which we left in white (see Table 4).

**Table 4:**
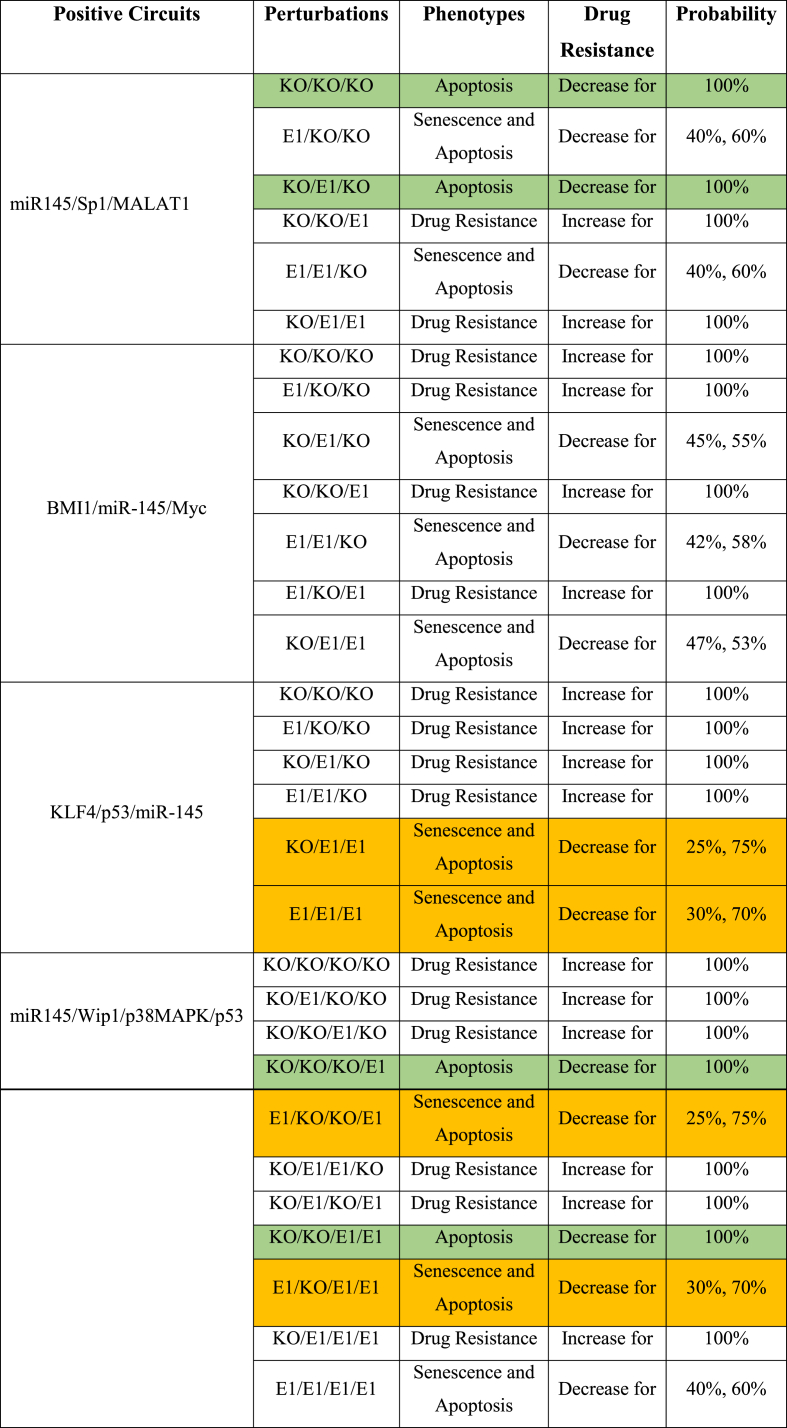
**Perturbations in the newly identified positive circuits**. Ectopic expression (E1) signifies gain-of-function (GoF), whereas knockdown (KO) reflects loss-of-function (LoF).

As we can see from the perturbations of the circuit between (miR-145/Sp1/MALAT1), overexpression of MALAT1 alone, or when combined with miR-145, causes drug resistance. Upregulation of Sp1 (E1) promotes apoptosis independently. Upregulation of miR-145 (E1) alone or in combination with Sp1 (E1), induces senescence and apoptosis. When all of the molecules are knocked out (KO/KO/KO), apoptotic cell death occurs. When we disrupted the following positive circuit (BMI1/miR-145/Myc), we discovered that overexpression (E1) of BMI1 alone or in conjunction with Myc (E1) generates drug resistance, whereas overexpression (E1) of BMI1 in combination with miR-145 (E1) induces senescence and apoptosis. Overexpression of miR-145, either alone or in combination with BMI1 (E1) or Myc (E1), causes senescence and apoptosis. The knockdown of miR-145, on the other hand, solely results in drug resistance. The other positive circuit between (KLF4/p53/miR-145) results in four cases of drug resistance, notably, knockdown (KO) of miR-145, whereas overexpression of miR-145 in conjunction with overexpression of p53 triggers senescence and apoptosis. More precisely, when miR-145 is activated (whether KLF4 or p53 is activated or shut down), senescence and apoptosis occur. Ultimately, when the miR-145/Wip1/p38MAPK/p53 positive circuit was disrupted, all situations of p53 knockdown (KO) resulted in drug resistance, whereas p53 overexpression (E1) alone or in association with p38MAPK overexpression (E1) resulted in apoptosis. Overexpression (E1) of p53 in conjunction with miR-145 (E1) and p38MAPK overexpression (E1) triggers senescence and apoptosis.

Furthermore, we exclusively employed Monte Carlo simulations (10.000 runs) in bistability scenarios, i.e., when senescence and apoptosis occur together (two probabilities in Table 4). This enables us to distinguish between bistability scenarios that favor drug resistance and those that oppose it. Thus, we expected that when the apoptotic phenotype exceeds 70%, these bistability scenarios result in lowering the drug resistance, which we showed in orange cells as shown in Table 4. The remaining bistability cases, where the apoptotic phenotype cannot surpass 70%, may contribute to improved drug resistance, and we have left them in white. Moreover, during perturbation analysis of the three positive circuits (miR-145/Sp1/MALAT1, KLF4/p53/miR-145, and miR-145/Wip1/p38MAPK/p53), oscillations were seen only in a few cases. We decided not to analyze them due to their association with cyclic attractors, which may be linked with cells escaping cycle arrest as suggested by Reyes et al. [51] and Sarin et al. [52]; thus, they are included in Supplementary Table S2.

Our detailed circuit analysis has unveiled four novel positive circuits intricately involved in drug resistance in NSCLC. These circuits comprise miR-145/Sp1/MALAT1, BMI1/miR-145/Myc, KLF4/p53/miR-145, and miR-145/Wip1/p38MAPK/p53. Within these circuits, miR-145 plays a central role, orchestrating interactions with essential transcription factors and proteins such as Sp1, Myc, Wip1, and KLF4, alongside MALAT1 and BMI1. The profound interactions within these circuits offer valuable insights into potential mechanisms governing drug resistance and influencing crucial cell fate decisions in NSCLC. These significant findings emphasize the utmost importance of delving into the intricate regulatory networks involving miR-145 and its associated molecules, holding promising potential as targets to overcome drug resistance in NSCLC.

## 4 Discussion

We used a systems biology approach to develop a Boolean model for cancer stemness and drug resistance in NSCLC. We established our Boolean model considering existing research publications (see Fig. 1). It is well known that Myc [53], Sp1 [54], BMI1 [54], and MALAT1 [1] are cancer stem cell (CSC) markers implicated in chemoresistance in NSCLC. MiR-145, on the other hand, inhibits CSCs while improving drug sensitivity [2]. According to new research, MALAT1 is upregulated in NSCLC and directly targets miR-145, which promotes KLF4 activation and drug resistance [1]. Another piece of evidence comes from a study by Chang et al. [2], who revealed that miR-145 is downregulated when BMI1 is overexpressed, which boosts Sp1 expression and accelerates EMT in pemetrexed-resistant NSCLC cells [2]. Interestingly, Sp1 controls MALAT1 expression in NSCLC [3]. Furthermore, miR-145 is down-regulated in NSCLC, and it was shown that overexpression of miR-145 can limit NSCLC development by inhibiting Myc [4] and Bcl2 [5], resulting in cell cycle arrest/senescence or apoptotic cell death. We evaluated our model, inspired by the papers stated above, to see if it might achieve comparable findings to those obtained in these studies [1,2,4,5]. As seen in Fig. 2, our model performs nicely with these investigations.

Furthermore, biochemical circuits play a significant role in GRNs [22]. GRNs are, in fact, a mixture of positive and negative circuits [21]. These circuits can acquire and interpret the biological system's dynamics [12,13,21,55], see Table 2. We found four novel positive circuits in this setting (miR-145/Sp1/MALAT1, BMI1/miR-145/Myc, KLF4/p53/miR-145, and miR-145/Wip1/p38MAPK/p53). We also gave evidence of these circuits based on how they interact in NSCLC (for more details see Table 3). Additionally, we conducted perturbation analyses on each circuit component, as detailed in Table 4. The circuit perturbation analysis revealed that in most scenarios, these circuits were associated with an increase in drug resistance. However, interestingly, we also identified instances in which perturbations led to the reduction of drug resistance (see Table 4, highlighted in green and orange cells). These findings underscore the complexity of the regulatory networks involved in drug resistance in NSCLC and suggest the potential for targeting specific components within these circuits as promising therapeutic strategies to overcome drug resistance.

MALAT1 and BMI1 are potent inhibitors of miR-145 activity, whereas Sp1, Myc, KLF4, and Wip1 are targeted by miR-145. Our findings imply that MALAT1 and BMI1 can positively regulate Sp1, Myc, KLF4, and Wip1 by targeting miR-145, hence accelerating drug resistance. Moreover, new data shows that Wip1 is implicated in drug resistance in NSCLC via targeting p38MAPK [56]. Interestingly, Wip1 is the direct target of miR-145 [50]. Additionally, previously Takekawa et al. [48] explored the negative circuit between p53/Wip1/p38MAPK in NSCLC. In more detail, Takekawa and colleagues [48] found that Wip1 plays a role in the control of the p38-p53 signaling pathway through a negative feedback mechanism since Wip1 expression is elevated in response to UV radiation by a process that necessitates both p38MAPK and p53. Furthermore, Takekawa et colleagues [48] demonstrated that p53 stimulates Wip1 expression, which inhibits p53 phosphorylation by inhibiting p38MAPK, establishing a negative circuit in DDR. It's interesting to note that, in contrast to Takekawa et al. [48], we found (a double negative) positive circuit between miR-145/Wip1/p38MAPK/p53. In fact, miR-145 is a novel component of the negative circuit described by Takekawa et al. [48] in NSCLC. The p53 induces miR-145 expression, which promotes p38MAPK expression by targeting Wip1, and activated p38MAPK stimulates p53 expression, resulting in a positive circuit. Collectively, our results suggest that miR-145 could serve as a system initialization for modulating drug resistance in NSCLC. Furthermore, according to the Drug Trial database (https://clinicaltrials.gov/), [57], miR-145 is a potential candidate for intervention against a broad spectrum of diseases, including cancer.

**Fig. 3:**
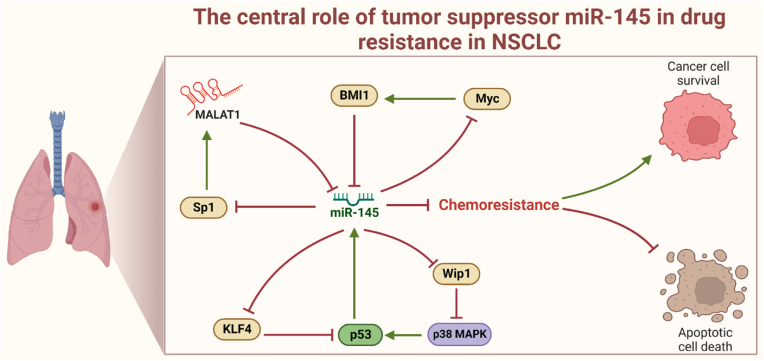
**The central role of miR-145-5p in the drug resistance mechanism within NSCLC.** Our significant findings reveal novel regulatory circuits involving miR-145. Firstly, miR-145 suppresses Myc, an inducer of BMI1, creating a positive circuit miR-145/Myc/BMI1. Secondly, miR-145 inhibits Sp1, a positive regulator of MALAT1, forming an additional positive circuit miR-145/Sp1/MALAT1. Moreover, miR-145 represses KLF4, leading to p53 inhibition, resulting in a positive circuit miR-145/KLF4/p53. Lastly, miR-145 negatively regulates Wip1, increasing p38 MAPK activity, which, in turn, upregulates p53 expression, amplifying miR-145 and forming the positive circuit miR-145/Wip1/p38MAPK/p53. These circuits illuminate the complex interactions governing drug resistance pathways in NSCLC, highlighting the pivotal role of miR-145-5p.

Despite the significant progress in understanding these circuits, some aspects remain unexplored and warrant further investigation. Our findings suggest that these novel circuits could play a crucial role in regulating drug sensitivity in NSCLC. Therefore, it is imperative to conduct in-depth investigations into the precise molecular pathways through which miR-145 influences drug resistance in NSCLC. Given the complexity of drug resistance mechanisms, other lncRNAs or miRNAs may also be pivotal in controlling drug resistance in NSCLC. Nonetheless, our results underscore the significance of miR-145 in drug resistance in NSCLC (see Fig. 3). However, it's important to note that our method's limitations include its inability to accurately predict time-dependent capabilities and the precise evolution of expression levels over time. To validate the functional relevance and impact of these circuits on drug resistance in NSCLC, future studies should prioritize experimental validation, both in vitro and in vivo. Furthermore, exploring the interplay between these circuits and other established drug resistance pathways could provide a more comprehensive understanding of the overarching regulatory network governing drug resistance in NSCLC.

In summary, our dynamic Boolean network, based on clinical data and experimental observations, reveals novel positive circuits: miR-145/Sp1/MALAT1, BMI1/miR-145/Myc, KLF4/p53/miR-145, and miR-145/Wip1/p38MAPK/p53, all contributing to drug resistance in NSCLC. MiR-145 emerges as a central regulator within these circuits, emphasizing its pivotal role in NSCLC drug resistance. Thus, our findings support that targeting MALAT1 and BMI1 shows potential for overcoming drug resistance, while activating miR-145 may reduce drug resistance in NSCLC.

## Data availability

All data needed to evaluate the conclusions in the paper are present in the paper and/or the Supplementary Materials.

## Code Availability

The model code is available in the GitHub repository: (https://github.com/GuptaShan/Dynamic-BNM-Drug-Resistance.git)

## CRediT authorship contribution statement

**Shantanu Gupta:** Conceptualization, Data curation, Formal analysis, Funding acquisition, Investigation, Methodology, Project administration, Resources, Software, Validation, Visualization, Writing – original draft, Writing – review & editing. **Daner A. Silveira:** Conceptualization, Data curation, Formal analysis, Investigation, Methodology, Visualization, Writing – original draft, Writing – review & editing. **Gabriel P.S. Piedade:** Conceptualization, Data curation, Formal analysis, Resources, Visualization. **Miguel P. Ostrowski:** Conceptualization, Data curation, Formal analysis, Resources, Visualization. **José Carlos M. Mombach:** Conceptualization, Data curation, Formal analysis, Investigation, Methodology, Project administration, Resources, Software, Validation, Visualization, Writing – review & editing. **Ronaldo F. Hashimoto:** Conceptualization, Data curation, Formal analysis, Funding acquisition, Investigation, Methodology, Project administration, Resources, Software, Supervision, Validation, Visualization, Writing – original draft, Writing – review & editing.

## Declaration of competing interest

The authors declare that they have no known competing financial interests or personal relationships that could have appeared to influence the work reported in this paper.

## Appendix A Supplementary data

Supplementary Table S1 Logical rules that control the node activity. Supplementary Table S1

Supplementary Table S2 Oscillations perturbation of the positive circuits. Supplementary Table S2
