# Supplementary material for: A dynamic Boolean network reveals that the BMI1 and MALAT1 axis is associated with drug resistance by limiting miR-145-5p in non-small cell lung cancer
Source: Noncoding RNA Res. 2023 Oct 19;9(1):185–93. doi: 10.1016/j.ncrna.2023.10.008 (PMC10730431; doi:10.1016/j.ncrna.2023.10.008)
Supplement: Supplementary Table S1 — Logical rules that control the node activity. [file mmc1.pdf]

Supplementary Table S1

Table S1: Logical rules that control node states of the model in Fig. 1 (highlighted in Blue) in terms of the state of its regulators. The left-hand side lists the official names of the molecules (target node) (highlighted in cream colour), while the right-hand side lists the references of each upcoming interaction to the target node.

Official names of the molecules	Target node	Interactions	Descriptions	References
**ATM serine/threonine kinase**	ATM	DNA_Damage	DNA damage directly activates ATM	PMID: 23939379
		HDAC1	HDAC1 directly inhibits ATM	PMID: 23939379
		E2F1	E2F1 directly activates ATM	PMID: 11459832
		BMI1	BMI1 directly inhibits ATM	PMID: 21383063
		Wip1	Wip1 directly inhibits ATM	PMID: 16949371
	Rule	DNA_Damage *AND* (*NOT* HDAC1 *OR NOT* Wip1 *OR* E2F1 *OR* BMI1)		ATM can be activated in the presence of DNA Damage and in the absence of HDAC1 or in the absence of WIP1 or in the absence of BMI1 or in the presence of E2F1.
**Mitogen-activated protein kinase 14**	p38MAPK	ATM	ATM directly activates p38MAPK	PMID: 21570395
		Wip1	Wip1 inhibits p38MAPK	PMID: 32296033
	Rule	ATM *AND NOT* Wip1		p38MAPK can be activated in the presence of ATM and the absence of Wip1
**microRNA-145-5p**	miR-145-5p	p53	p53 transactivates miR-145	PMID: 19202062
		MALAT1	MALAT1 inhibits miR-	PMID: 30250547

			145 expression	
		BMI1	BMI1 inhibits miR-145 expression	PMID: 36499676
	Rule	p53 *AND NOT* MALAT1 *AND NOT* BMI1		miR-145 can be activated in the presence of p53 AND in the absence of MALAT1 and in the absence of BMI1.
**Specificity protein 1**	Sp1	BMI1	BMI1 induces Sp1 expression	PMID: 36499676
		Myc	Myc induces Sp1 expression	PMID: 17264126
		miR-145	miR-145 inhibits Sp1 expression	PMID: 36499676
	Rule	(BMI1 *AND* Myc) *OR NOT* miR145		Sp1 can be activated in the presence of BMI1 AND Myc Or in the absence of miR-145
**Metastasis-associated lung adenocarcinoma transcript 1**	MALAT1	Sp1	Sp1 induces MALAT1 expression	PMID: 25773124
	Rule	Sp1		MALAT1 can be activated in the presence of Sp1.
**B lymphoma Mo-MLV insertion region 1 homolog**	BMI1	Myc	Myc induces BMI1 expression	PMID: 10541554
		E2F1	E2F1 induces BMI1 expression	PMID: 16582100
	Rule	Myc *OR* E2F1		BMI1 can be activated in the presence of E2F1 OR Myc.
**Krüppel-like Factor 4**	KLF4	miR-145	miR-145 inhibits KLF4 expression	PMID: 30250547
		E2F1	E2F1 induces KLF4	PMID: 28068326

		HDAC1	HDAC1 inhibits KLF4	PMID: 25341045
		p53	p53 induces KLF4	PMID: 12427745
	Rule	*NOT* miR145 *OR* (E2F1 *AND NOT* HDAC1 *AND* p53)		KLF4 can be activated in the presence of E2F1 or p53 and in the absence of miR-145 or in the absence of HDAC1.
**HDAC1**	HDAC1	Sirt_1	Acetylation of HDAC1 and degradation of SIRT1	PMID: 25950477
		DNA_Damage	DNA Damage directly inhibits HDAC1	PMID: 23939379
	Rule	*NOT* Sirt_1 *AND NOT* DNA_Damage		HDAC1 can be activated in the absence of Sirt1 AND in the absence of DNA Damage.
**Myc proto-oncogene protein**	Myc	E2F1	E2F1 directly activates Myc	PMID: 18345030
		miR-145	Myc is a direct target of miR-145	PMID: 19202062
		RB	RB directly inhibits Myc expression	PMID: 27105536
		p21	p21 directly inhibits Myc	PMID: 16923815
		p38MAPK	p38MAPK can activates Myc	PMID: 16365184
	Rule	(E2F1 *OR* p38MAPK *OR NOT* p21) *AND NOT* RB *AND NOT* miR-145		Myc can be activated in the presence of E2F1 or the presence of p38MAPK or the absence of miR-145 and the absence of RB and p21.

**Tumor suppressor p53 protein**	p53	ATM	ATM activates p53 transcription	PMID: 11526498
		p38MAPK	p38MAPK activates p53	PMID: 15642743
		HDAC1	HDAC1 inhibits p53	PMID: 31096697
		KLF4	KLF4 inhibits p53	PMID: 16244670
		MALAT1	MALAT1 inhibits p53 expression	PMID: 29632545
		Mdm2	Mdm2 inhibits p53	PMID: 12563309
	Rule	*(*ATM *AND NOT* KLF4*) OR (NOT* Mdm2 *AND* p38MAPK *AND NOT* HDAC1 *AND NOT* MALAT1*)*		p53 can be activated in the presence of ATM or the absence of MDM2 and the presence of p38MAPK and the absence of HDAC1 or in the absence of KLF4 and MALAT1.
**E3 ubiquitin protein ligase homolog protein**	Mdm2	Wip1	Wip1 negatively regulates Mdm2	PMID: 17936559
		p53	p53 activates Mdm2	PMID: 12563309
		miR-145	miR-145 inhibits Mdm2 expression	PMID: 22330136
		ATM	ATM inhibits Mdm2	PMID: 16082221
	Rule	(*NOT* Wip1 *OR* p53) *AND NOT* ATM *AND NOT* miR-145		Mdm2 can be activated in the presence of p53 or the absence of Wip1 and the absence of ATM or in the absence of miR-145.
**Tumor suppressor p53 protein (Ser-15 and Ser-20)**	p53_A	Sirt_1	Sirt1 inhibits p53	PMID: 20471503
		p53	p53 phosphorylate	PMID: 21576488

			d at Ser-15 and Ser-20	
		p53_K	p53 phosphorylated at Ser-46 inhibits Ser-15 and Ser-20	PMID: 21576488
		p53INP1	Control of p53 accumulation	PMID: 21576488
	Rule	*NOT* Sirt_1 *AND NOT* p53_K *AND* (p53:1 *OR NOT* p53_INP1)		p53_A can be activated in the absence of Sirt1 and the absence of p53_K and the presence of p53 or the absence of p53_INP1.
**Tumor suppressor p53 protein (Ser-46)**	p53_K	Sirt_1	Sirt1 inhibits p53	PMID: 20471503
		p53_A	p53 phosphorylated at Ser-46	PMID: 21576488
		p53	p53 phosphorylated at Ser-15 and Ser-20 inhibits Ser-46	PMID: 21576488
		Wip1	Wip1 inhibits p53	PMID: 27959454
	Rule	*NOT* p53_A *AND* (*NOT* Sirt_1 *OR NOT* Wip1) *AND* p53		p53_K can be activated in the absence of p53_A and the absence of Sirt1 or the absence of Wip1 and the presence of p53.
**Sirtuin 1**	Sirt_1	E2F1	E2F1 activates Sirt1	PMID: 24020005
		miR-145	Sirt1 is a directly target of miR-145	PMID: 32852406
		HDAC1	HDAC1 inhibits Sirt1	PMID: 18193082
	Rule	E2F1 *AND NOT* miR-145 *AND NOT* HDAC1		Sirt1 can be activated in the presence of E2F1 and the absence of

				miR-145 and HDAC1.
**Tumor protein p53 inducible nuclear protein 1**	p53_INP1	p53_A	p53 Ser-15 and Ser-20 activate p53-INP1	PMID: 21576488
		p53_K	p53 Ser-46 activates p53-INP1	PMID: 21576488
	Rule	p53_A *OR* p53_K		p53_INP1 can be activated in the presence of p53_A or p53_K.
**Protein Phosphatase 1D**	Wip1	p53_A	p53 activates Wip1	PMID: 26883196
		miR-145	miR-145 inhibits Wip1	PMID: 34899906
	Rule	p53_A *AND NOT* miR-145		Wip1 can be activated in the presence of p53_A and in the absence of miR-145.
**Cyclin-dependent kinase inhibitor 1A**	p21	p53_A	p53 activates p21	PMID: 31416295
		HDAC1	Repression of HDAC1 activates p21 expression	PMID: 26794658
		Myc	Repression of Myc activates p21 expression	PMID: 27105536
		p38MAPK	p38MAPK can activates p21	PMID: 19528229
		Caspase3	Caspase3 inhibits p21	PMID: 10022118
		BMI1	BMI1 inhibits p21	PMID: 24552182
	Rule	p53_A *OR* (*NOT* (HDAC1 *OR* Myc) *AND NOT* BMI1 *AND NOT* Caspase3 *AND* p38MAPK)		p21 can be activated in the presence of p53-A or the absence of HDAC1 or BMI1 and the absence of Caspase3 and the presence of p38MAPK.

**Cell division cycle 25A**	Cdc25A	ATM	ATM directly inhibits Cdc25A	PMID: 22263797
		p38MAPK	p38MAPK inhibits Cdc25A expression	PMID: 22263797
	Rule	*NOT* ATM *AND NOT* p38MAPK		Cdc25A can be activated in the absence of ATM and in the absence of p38MAPK.
**Cyclin-dependent kinases 4 and 6 complex/Cyclin D1**	CDK46_CycD	Cdc25A	Cdc25A activates CDK46-CycD	PMID: 28192398
		p21	p21 directly inhibits CDK46-CycD	PMID: 21616632
		miR-145	CDK46-CycD is a direct target of miR-145	PMID: 28051259
	Rule	Cdc25A *AND NOT* miR_145 *AND NOT* p21		CDK46_CycD can be activated in the presence of Cdc25A and the absence of miR-145 and the absence of p21.
**Cyclin-dependent kinase 2/CyclinE2**	CDK2_CycE	Cdc25A	Cdc25A activates CDK2-CycE	PMID: 22263797
		E2F1	E2F1 Activates CDK2-CycE	PMID: 29754146
		p21	p21 directly inhibits CDK2-CycE	PMID: 21616632
	Rule	Cdc25A *AND* E2F1 *AND NOT* p21		CDK2_CycE can be activated in the presence of Cdc25A and the presence of E2F1 and the absence of p21.
**Retinoblastoma 1 protein**	RB	CDK46_CycD	CDK46-CycD directly inhibits RB	PMID: 12362273

		CDK2_CycE	CDK2-CycE directly inhibits RB	PMID: 12362273
	Rule	*NOT* CDK46_CycD *AND NOT* CDK2_CycE		RB can be activated in the absence of CDK46_CycD and CDK2_CycE.
**BCL2 binding component 3**	PUMA	p53_K	PUMA is activated by p53-K	PMID: 19641508
	Rule	p53_K		PUMA can be activated in the presence of p53_K.
**BCL2 apoptosis regulator**	BCL2	PUMA	PUMA directly inhibits BCL2	PMID: 30250075
		miR-145	BCL2 is a direct target of miR-145	PMID: 29541201
	Rule	*NOT* PUMA *AND NOT* miR-145		BCL2 can be activated in the absence of PUMA and the absence of miR-145.
**BCL2 associated X, apoptosis regulator**	BAX	BCL2	BCL2 directly inhibits BAX	PMID: 23173842
		KLF4	KLF4 inhibits BAX	PMID: 23162646
		p53_K	p53-k activates BAX	PMID: 14667504
	Rule	(*NOT* BCL2 *OR NOT* KLF4) *AND NOT* p53_K		BAX can be activated in the absence of BCL2 or in the absence of KLF4 and the presence of p53_K.
**E2F transcription factor 1**	E2F1	RB	RB directly inhibits E2F1	PMID: 23967231
		Cdc25A	Cdc25A activates E2F1 expression	PMID: 22263797
		ATM	ATM activates E2F1	PMID: 11459832
		Sirt_1	Sirt1 inhibits E2F1	PMID: 24020005

		MALAT1	MALAT1 directly induces E2F1 expression	PMID: 36690318
		Myc	Myc activates E2F1 expression	PMID: 17784791
	Rule	(*NOT* RB *AND* ((Cdc25A *AND* ATM) *OR NOT* Sirt_1)) *OR* MALAT1 *OR* Myc		E2F1 can be activated in the absence of RB and the presence of Cdc25A and the presence of ATM or the absence of Sirt1 or the presence of MALAT1 or the presence of Myc.
**Caspase-3**	Caspase3	BCL2	BCL2 inhibits Caspase3	PMID: 16297711
		p21	p21 inhibits Caspase3	PMID: 19440234
		BAX	BAX enhances Caspase3 activity	PMID: 26395559
	Rule	*NOT* (BCL2 *AND* p21) *AND* BAX		Caspase3 can be activated in the absence of BCL2 and the absence of p21 and the presence of BAX.
**Proliferation**	Proliferation	E2F1	E2F1 induces Proliferation	PMID: 23210897
		p53	p53 inhibits proliferation in NSCLC	PMID: 31737176
	Rule	CDK2_CycE *AND NOT* p53		CDK2_CycE induced tumorigenesis and progression in cancer cells.
**Drug Resistance**	Drug Resistance	MALAT1	MALAT1 induces Drug resistance	PMID: 29505924
		RB	RB induces Drug resistance	PMID: 35409416
	Rule	MALAT1 *AND* RB		

				Drug resistance can be activated when RB and MALAT1 were activated.
**Senescence**	Senescence	p21	p21 induces Senescence	PMID: 31204100
		p53_A	p53 A induces senescence	PMID: 32182711
		Myc	Myc inhibits Senescence	PMID: 30526305
	Rule	p21 *AND NOT* CDK2_CycE		Senescence can be activated in the presence of p21 and the absence of Myc
**Apoptosis**	Apoptosis	Caspase3	Caspase3 induces Apoptosis	PMID: 20368820
	Rule	Caspase3 induces Apoptosis in NSCLC		Caspase3 can activated Apoptosis in the cancer cells, in response to DNA Damage.
