# Supplementary material for: A dynamic Boolean network reveals that the BMI1 and MALAT1 axis is associated with drug resistance by limiting miR-145-5p in non-small cell lung cancer
Source: Noncoding RNA Res. 2023 Oct 19;9(1):185–93. doi: 10.1016/j.ncrna.2023.10.008 (PMC10730431; doi:10.1016/j.ncrna.2023.10.008)
Supplement: Supplementary Table S2 — Oscillations perturbation of the positive circuits. [file mmc2.pdf]

Supplementary Table S2

Table S2: Oscillations were observed during the perturbation analysis of these three positive circuits (miR-145/Sp1/MALAT1, KLF4/p53/miR-145, and miR-145/Wip1/p38MAPK/p53), which we have listed below.

**Positive Circuits**	**Perturbations**	**Phenotypes**
miR-145/Sp1/MALAT1	KO/KO/E1	Oscillations
	E1/E1/E1	
BMI1/miR-145/Myc	E1/E1/E1	
KLF4/p53/miR145	KO/KO/E1	
	E1/KO/E1	
miR-145/Wip1/p38MAPK/p53	E1/KO/KO/KO	
	E1/E1/KO/KO	
	E1/KO/E1/KO	
	E1/E1/E1/KO	
